# C-954-05. Burn Patients Who Leave Against Medical Advice: A Nationwide Analysis

**DOI:** 10.1093/jbcr/irag033.137

**Published:** 2026-04-06

**Authors:** Manuel Castillo-Angeles, Barbara U Okafor, Anupama Mehta

**Affiliations:** Brigham and Women's Hospital, Boston, MA; Brigham and Women's Hospital, Boston, MA; Brigham and Women's Hospital, Boston, MA

## Abstract

**Introduction:**

Discharge against medical advice (AMA) constitutes 1-2% of all hospital discharges, ultimately resulting in poorer health outcomes, the fragmentation of patient care, and increased healthcare costs. However, there is a paucity of studies focusing on patients with burn injuries. Our goal was to determine the prevalence and predictors of leaving AMA within the burn population.

**Methods:**

This was a retrospective analysis of the Nationwide Inpatient Sample (2015-2018). We included patients with a primary diagnosis of burn injury. We collected demographics and clinical characteristics. Analysis was restricted to patients who were either discharged home or discharged AMA. Multivariable logistic regression was used to identify risk factors associated with leaving AMA.

**Results:**

A weighted total of 109 570 burn injury admissions were identified. Mean age was 34.59 (SD 14.13), 38% were female, 54% were white, and 1925 (1.76%) were discharged AMA. After adjusted analysis, female sex (adjusted odds ratio [aOR] 0.72, 95% Confidence Interval [CI] 0.57 – 0.92) and Hispanic ethnicity (aOR 0.52, 95% CI 0.33 – 0.80) were associated with lower odds of being discharged AMA. However, age (aOR 1.01, 95% CI 1.01 – 1.02) and having public insurance (aOR 2.09, 95% CI 1.51 – 2.88) or being uninsured (aOR 3.97, 95% CI 2.72 – 5.81) were associated with a higher risk of leaving AMA.

**Conclusions:**

This study shows that race and insurance status are the most important determinants of being discharged AMA within the burn population. Although the prevalence of leaving AMA among burn patients is comparable to that observed in other conditions, future efforts should prioritize the early identification of these vulnerable patients.

**Applicability of Research to Practice:**

This study shows which factors are important predictors of burn patients leaving AMA, and therefore, these results can guide the development of tailored strategies that should be implemented to address these disparities and thereby prevent AMA discharges among this patient population.

**Funding for the study:**

N/A.

